# New insights in *Staphylococcus pseudintermedius* pathogenicity: antibiotic-resistant biofilm formation by a human wound-associated strain

**DOI:** 10.1186/s12866-015-0449-x

**Published:** 2015-05-21

**Authors:** Arianna Pompilio, Serena De Nicola, Valentina Crocetta, Simone Guarnieri, Vincenzo Savini, Edoardo Carretto, Giovanni Di Bonaventura

**Affiliations:** Department of Medical, Oral, and Biotechnological Sciences, “G. d’Annunzio” University, Via Vestini 31, Chieti, 66100 Italy; Center for Research on Ageing, “G. d’Annunzio” University Foundation, Chieti, Italy; Department of Neuroscience and Imaging, “G. d’Annunzio” University, Chieti, Italy; Clinical Microbiology and Virology, “Spirito Santo” Hospital, Pescara, Italy; IRCCS Arcispedale, “Santa Maria Nuova”, Reggio Emilia, Italy

**Keywords:** *Staphylococcus pseudintermedius*, zoonotic infection, Biofilm formation, Wound infection, Antibiotic-resistance

## Abstract

**Background:**

*Staphylococcus pseudintermedius* is an opportunistic pathogen recognized as the leading cause of skin, ear, and post-operative bacterial infections in dogs and cats. Zoonotic infections have also recently been reported causing endocarditis, infection of surgical wounds, rhinosinusitis, and catheter-related bacteremia. The aim of the present study is to evaluate, for the first time, the pathogenic potential of *S. pseudintermedius* isolated from a human infection. To this end, strain DSM 25713, which was recently isolated from a wound of a leukemic patient who underwent a bone marrow transplantation, was investigated for biofilm formation and antibiotic-resistance under conditions relevant for wound infection.

**Results:**

The effect of pH (5.5, 7.1, and 8.7) and the presence of serum (diluted at 1:2, 1:10, and 1:100) on biofilm formation was assessed through a crystal violet assay. The presence of serum significantly reduced the ability to form biofilm, regardless of the pH value tested. *In vitro* activity of eight antibiotics against biofilm formation and mature 48 h-old biofilms was comparatively assessed by crystal violet assay and viable cell count, respectively. Antibiotics at sub-inhibitory concentrations reduced biofilm formation in a dose-dependent manner, although cefoxitin was the most active, causing a significant reduction already at 1/8xMIC. Rifampicin showed the highest activity against preformed biofilms (MBEC_90_: 2xMIC). None of the antibiotics completely eradicated the preformed biofilms, regardless of tested concentrations. Confocal and electron microscopy analyses of mature biofilm revealed a complex “mushroom-like” architecture consisting of microcolonies embedded in a fibrillar extracellular matrix.

**Conclusions:**

For the first time, our results show that human wound-associated *S. pseudintermedius* is able to form inherently antibiotic-resistant biofilms, suggestive of its pathogenic potential, and consistent with recent reports of zoonotic infections.

## Introduction

*Staphylococcus pseudintermedius*, the prevalent species in the *Staphylococcus intermedius* Group, is an opportunistic pathogen recognized as the leading cause of skin, ear, and post-operative bacterial infections in dogs and cats [1, 2].

Human infection, mainly acquired from dogs, has however only recently been reported. The first case of human infection by *S. pseudintermedius* was described in 2006 by Van Hoovels *et al*. [3] causing endocarditis after the implantation of a cardioverter-defribrillator device (ICD). Since then, human infections have been reported sporadically, including surgical site infections, rhinosinusitis, and catheter associated bacteremia [4–6].

The last published case of human *S. pseudintermedius* infection, the second case involving a methicillin-resistant strain, recently arrived at our observation [7]. A 65-year-old male patient who received an allogeneic bone marrow transplant for chronic lymphoblastic leukemia, was admitted to the “Spirito Santo” Hospital of Pescara (Italy), because of a wound infection. The lesion, secondary to the chronic Graft-versus-Host Disease (GvHD) that complicated the transplant, was located in the periumbilical region and showed two different purulent discharges that grew *S. pseudintermedius*, namely strain DSM (Deutsche Sammlung von Mikroorganismen und Zellkulturen, GmbH) 25713. The patient had a history of close association with a companion dog, as well as farm cows.

Knowledge of the pathogenesis of *S. pseudintermedius* remains yet limited. It is known that veterinary strains are able to produce numerous virulence factors, including β-hemolysin, clumping factor, coagulase, DNase, protein A, lipase, leukotoxin, exfoliative toxin, and enterotoxins [8, 9]. Furthermore, *S. pseudintermedius* methicillin-resistant strains have recently emerged as a major challenge, for veterinary dermatologists in particular, owing to their extensive multidrug resistance and their behavior as nosocomial pathogens [10].

Biofilm formation is considered to be one of the most important virulence factors in staphylococci, especially for *Staphylococcus aureus* and *Staphylococcus epidermidis*, as it allows them to adhere to tissues and indwelling medical devices [11].

A biofilm is a structured consortium of bacteria adhered to a substratum and embedded in a self-produced extracellular polymer substance (EPS) consisting of polysaccharide, protein and DNA. Bacterial biofilms are of clinical relevance since they confer resistance to antibiotics and disinfectants, as well as resistance to phagocytosis and the host immune system generally, all factors promoting chronic infections.

Biofilm forming ability of veterinary *S. pseudintermedius* isolates has been reported, although not extensively [8, 12–14]. Most strains were identified as biofilm-producers [14], although isolates belonging to the most frequent sequence type observed in Europe, ST71, had a significantly greater ability to produce biofilm [12], with strains from canine conjunctivitis also demonstrating increased production [8].

Although the presence of virulence factors such as DNase, β-hemolysin, coagulase, and leukotoxins was also observed in *S. pseudintermedius* strains isolated from humans [3, 5], to the best of our knowledge the potential for biofilm formation of these strains has yet to be investigated.

Therefore, the present work was aimed at assessing, for the first time in literature, the ability of a human *S. pseudintermedius* strain to form biofilm, as well as its pathogenic potential. In this regard, biofilm formation by the wound isolate *S. pseudintermedius* strain DSM 25713 was evaluated under different conditions relevant for wound site (i.e. different concentrations of serum, tested as free or substratum-adsorbed; and different pH values suggestive of acid, neutral and basic wound environments), and in the presence of eight antibiotics tested at both sub-inhibitory and bactericidal concentrations against biofilm formation and preformed (mature) biofilms, respectively. Biofilm architecture and kinetics of formation were further studied using both scanning electron and confocal laser scanning microscopy.

Overall, our results clearly show that *S. pseudintermedius* strain DSM 25713 is able to form a biofilm ultrastructurally complex that is inherently resistant to antibiotics, confirming the pathogenic potential of this bacterium to cause human disease.

## Materials and methods

### Bacterial strain and growth conditions

The strain *S. pseudintermedius* DSM 25713 was isolated from a wound of a haematologic patient recently admitted to “Santo Spirito” Hospital in Pescara, Italy [7]. Strain identification was carried out using biochemical tests (API system; bioMerieux, Marcy l’Etoile, France), and confirmed by 16S RNA sequencing. Bacterial stocks were stored at −80 °C until their use, when they were thawed, inoculated into Trypticase Soy broth (TSB; Oxoid SpA, Garbagnate M.se, Italy), and incubated at 37 °C for 24 h. An aliquot was then plated twice on Mueller-Hinton agar (MHA; Oxoid SpA) to check for the purity of the culture. A standardized suspension of 1.0 × 10^8^ CFU/mL (corresponding to OD of 1.0 at 550 nm) was prepared in TSB and used immediately for all experiments.

### Standardization and optimization of *S. pseudintermedius* biofilm growth on polystyrene

Since the optimal conditions for *S. pseudintermedius* biofilm formation on polystyrene surfaces are not known, preliminary experiments were carried out to optimize and standardize the *in vitro* model for biofilm formation. The following basic parameters for biofilm growth were considered for optimization: i) inoculum size (suspensions at 10^5^, 10^6^, and 10^7^ CFU/ml were prepared starting from standardized inoculum); ii) dynamic (cultures were incubated under agitation at 200 rpm) (IKA agitator KS 260; IKA, Milan, Italy) or static conditions; and iii) incubation time (24, 48, and 72 h).

Based on our results, an inoculum size of 10^7^ CFU/ml, and static incubation were used for *S. pseudintermedius* biofilm formation, while susceptibility to antibiotics was tested by exposing 48 h-biofilms to antibiotic for a further 24 h.

### Quantitative measurement of static biofilms

In brief, 200 μl of the standardized inoculum at desired concentration prepared in TSB (Oxoid SpA) was added aseptically to each well of a 96-well polystyrene tissue culture plate (Falcon BD; Becton, Dickinson and Company, Milan, Italy), and incubated at 37 °C under static conditions. Wells that only contained TSB were considered as controls. At the end of the incubation, spent medium was discarded and each well was washed twice with PBS (pH 7.2) (Sigma-Aldrich Srl, Milan, Italy) to remove non-adherent cells. Biofilm formation was then assessed by crystal violet assay or viable cell count. i) Crystal violet microtiter plate assay [15]. Biofilm samples were fixed by incubating plates at 60 °C for 1 h, then stained for 5 min with 200 μl Hucker-modified crystal violet [16]. Excess stain was rinsed off with running tap water, and then the plates were air-dried. Crystal violet was extracted by exposure at room temperature for 15 min to 200 μl glacial acetic acid 33 % (Sigma-Aldrich), and biofilm biomass (including adherent bacteria and EPS) was then assessed by measuring the optical density at 492 nm (OD_492_) (SpectraMax 190; Molecular Devices, Sunnyvale, CA, USA). ii) Total viable cell count. In each well, the biofilm sample was scraped by using a pipette tip after 5-min exposure to 200 μl trypsin-EDTA 0.25 % (Sigma-Aldrich), then resuspended in sterile PBS by vortexing. Serial 10-fold dilutions of each sample were prepared in sterile PBS and 100 μl of each dilution was plated on MHA and incubated at 37 °C for 24 h. Colonies were counted to estimate biofilm viability.

### Continuous flow through biofilm

Biofilm was allowed to form in a polycarbonate flow through chamber (The Technical University of Denmark, Lyngby, Denmark) for microscopic studies [17]. The flow cell is composed of three parallel channels in perspex (poly[methyl methacrylate]), covered with a no. 1 24 × 50 mm glass coverslip which serves as the biofilm substratum. Each channel has a dimension (length × width × height) of 40 × 4 × 4 mm and was cleaned with 96 % (v/v) ethanol prior to use.

In brief, the chamber was inoculated with standardized inoculum diluted in TSB at 5 × 10^5^ CFU/ml, then inverted to allow microorganisms to attach for 3 h, under static conditions, at 37 °C. The flow cell was then placed upright and the pump started with a TSB flow rate of 0.5 ml/min. Biofilm was allowed to form for 24 h at 37 °C, then washed with PBS (2 min at 0.5 ml/min), and finally observed by a confocal laser scanning microscope.

### Time course of biofilm formation

Biofilms were allowed to form in each well of a 24-well flat-bottom polystyrene tissue-treated microtiter plate (BD Company), as described above. At selected times (30 min, 1, 2, 4, 8, 24, 48, and 72 h of incubation) biofilm viability was assessed by viable colony count as described above. In a parallel series of experiments, wells were broken and fragments representative of each time point were observed by scanning electron microscopy.

### Effect of human serum and pH on biofilm formation

Serum for testing was pooled from multiple samples. Serum samples were collected from 30 blood donors, which were selected based on their health status as non-smokers with no other known current diseases, and because they were not on any medications. The serum samples were then pooled, aliquoted, and stored at −20 °C until use. Since it was observed that albumin and total protein levels were significantly higher in serum than in wound fluid [18], serum was tested against biofilm formation at different dilutions (1:2, 1:10, and 1:100) prepared in TSB. Serum was tested both as free (soluble) and adhered to polystyrene. In the latter case, serum-coated microplates were prepared immediately before use. In brief, 200 μl of serum was added to each well of a 96-well tissue culture plates (BD Company), incubated for 2 h at 37 °C, then washed by PBS to remove excess serum.

The effect of pH and serum on *S. pseudintermedius* biofilm formation was simultaneously assessed. To this end, 96-well microtiter plates containing free or adsorbed serum were inoculated with the standardized inoculum prepared in TSB that was previously corrected at different pH values (5.5, 7.1, and 8.7) by using HCl or NaOH 1 M solution, then incubated at 37 °C for 24 h. Biofilm biomass levels were then spectrophotometrically measured as described above.

### Susceptibility assays

Susceptibility of *S. pseudintermedius* strain DSM 25713 to chloramphenicol, gentamicin, cefoxitin, linezolid, rifampicin, vancomycin, tetracycline, and tigecycline (all were purchased, as reference powders, from Sigma-Aldrich) was determined by microdilution technique, in accordance with CLSI M100-S20 guidelines [19]. MIC was calculated as the lowest concentration of the test agent that completely inhibited visible growth. MBC was evaluated as the lowest concentration of the test agent killing of at least 99.99 % of the original inoculum. *E. faecalis* ATCC29212 and *E. coli* ATCC25922 were used as reference strains.

### Antibiotic activity against biofilm formation

In each well of a 96-well flat-bottom polystyrene tissue-culture microtiter plate (Becton, Dickinson and Company), 5 μl of a standardized inoculum (1–5 × 10^7^ CFU/ml) were added to 100 μl of cation-adjusted Mueller-Hinton broth (CAMHB; Oxoid SpA) containing antibiotic at 1/2×, 1/4×, and 1/8×MIC. After incubation at 37 °C for 24 h, non-adherent bacteria were removed by washing twice with 100 μl sterile PBS, then biofilm levels were spectrophotometrically measured as described above.

### Antibiotic activity against preformed biofilms

The activity of antibiotics against 48 h-old biofilms was assessed by viable colony count. Biofilms were allowed to form in each well of a 96-well flat-bottom polystyrene tissue-treated microtiter plate (Becton, Dickinson and Company), as described above. Following 48 h-incubation, biofilms samples were washed twice with PBS, then exposed to 200 μl of drug-containing CAMHB (prepared at 1, 2, 4, 8, 16, 32, 64, and 128  × MIC). After incubation at 37 °C for 24 h, non-adherent bacteria were removed by washing twice with 200 μl sterile PBS, and biofilm samples were scraped as described above. Cell suspension was then vortexed for 1 min to break up bacterial clumps. Bacterial counts were performed by plating serial 10-fold dilutions of this suspension on MHA plates. Control biofilm samples were not exposed to antibiotics. Minimum Biofilm Eradication Concentration (MBEC) was calculated as the minimum concentration of tested antibiotic able to eradicate preformed biofilm.

### Microscopic analyses

Kinetics of biofilm formation by *S. pseudintermedius* strain DSM 25713 and its architecture were assessed by scanning electron microscopy (SEM) and environmental-SEM (ESEM), respectively. The effects of exposure to several gentamicin concentrations as well as the ultrastructure of biofilm formed under dynamic incubation were evaluated by confocal laser scanning microscopy (CLSM). i) SEM and ESEM assays. Biofilm formation kinetics in TSB was monitored - under static conditions, without serum, at 37 °C, and at pH 7.1 - in 35 mm-tissue culture polystyrene dish (Becton, Dickinson and Company) at different time periods (30 min, 1, 2, 4, 8, 24, 48, and 72 h). Samples were then fixed in a mixture of 2 % paraformaldehyde (Electron Microscopy Sciences, Hatfield, PA, USA) + 2 % glutaraldehyde (Sigma-Aldrich) [vol/vol] in 0.15 M sodium cacodylate buffer (pH 7.4; Fluka), with 0.1 % alcian blue (Sigma-Aldrich). Samples were post-fixed for 90 min at room temperature in 1 % OsO4 [vol/vol] (Electron Microscopy Sciences) in 0.15 M cacodylate buffer, then dehydrated in an ascending ethanol series (50, 70, 80, 95, and twice 100 %; 10 min/each), dried for 30 min with hexamethyldisilazane (Polysciences Inc., Warrington, PA, USA), and finally air-dried. Specimens were coated with gold-palladium by Polaron E5100 II (Polaron Instruments Inc.), and then observed with a Philips XL30CP scanning electron microscope in the high-vacuum mode at 15 kV. In a parallel experiment, a 72 h-old biofilm sample was fixed and post-fixed as described above, and directly observed using a Zeiss EVO (Carl Zeiss SpA, Arese, Milan, Italy). ii) CLSM assay. Briefly, 48 h-biofilms were allowed to grow on polystyrene as described for SEM analysis, then exposed to gentamicin at different concentrations (from 1x to 128xMIC) for a further 24 h. Untreated biofilms were used as controls. In a parallel series of experiments, biofilm was allowed to grow under dynamic conditions in a flow cell system in the absence of antibiotics, as described above. Both static and flow cell biofilms were stained with Live/Dead BacLight kit (Molecular Probes Inc., Eugene, USA) and Concanavalin A (Alexa Fluor 647 coniugate; Molecular Probes Inc.). Static biofilm samples formed on polystyrene were placed in an Attofluor cell-chamber (Molecular Probes Inc.) before observation. CLSM analysis was performed with an LSM 510 META laser scanning microscope attached to an Axioplan II microscope (Zeiss Italia, Arese, Milan, Italy). Depth measurements were taken at regular intervals across the width of the device. To determine biofilm structure, a Z-series of 25 optical planes at xy resolution of 512×512 pixel (68.4 × 68.4 μm) with a thickness of 1.00 μm was taken throughout the biofilm. Both SEM and CLSM representative images were captured and processed for display using Photoshop (Adobe Systems Inc., San Jose, California) software.

### Statistical analysis and biofilm interpretative criteria

All experiments were carried out at least in triplicate and repeated at least on two different occasions. Differences were assessed by unpaired-*t* test (standardization of *in vitro* model of biofilm formation), ANOVA + Newman-Keuls multiple comparison post-test (effect of serum and pH both on biofilm formation and bacterial growth), chi-square test (percentage of reduction of both biofilm biomass formation and biofilm viability), or Kruskall-Wallis + Dunn’s multiple comparison post-test (kinetic of biofilm formation). Statistical analysis of results was conducted with GraphPad Prism version 6.00 (GraphPad software Inc.; San Diego, CA, USA), considering as statistically significant a *p* value of < 0.05.

The low cut-off value for biofilm formation was represented by 3 SDs above the mean OD_492_ of control wells (containing bacteria-free medium) [20].

To evaluate the effect of serum and pH on biofilm formation, biofilm levels were normalized for bacterial growth by calculating the specific biofilm formation (SBF) index: SBF = (OD_biofilm_ - OD_NC_)/OD_growth_ in which OD_biofilm_ is the OD_492_ of the stained biofilm, OD_NC_ is the OD_492_ of the stained negative control wells (to eliminate unspecific or abiotic OD values), and OD_growth_ is the OD_600_ of cells grown in broth.

The percentage of inhibition of biofilm formation by antibiotics tested at sub-inhibitory concentrations was calculated as follows: (1 - OD_exp_/OD_UC_) × 100 in which OD_exp_ is the OD_492_ of the stained antibiotic-exposed biofilm, and OD_UC_ is the OD_492_ of the stained untreated control biofilm.

## Results

### Standardization and optimization of biofilm growth

**Fig. 1:**
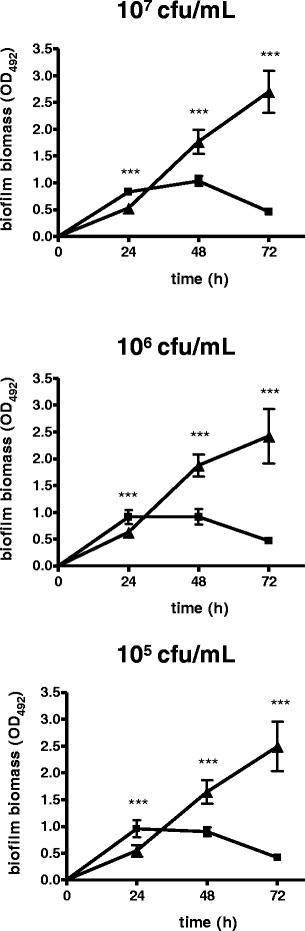
Standardization of experimental conditions for biofilm formation by *S. pseudintermedius* strain DSM 25713 on polystyrene surface. Effect of dynamic (filled squares) or static (filled triangles) incubation, incubation time (24, 48, and 72 h), and inoculum concentration (10^5^, 10^6^, and 10^7^ CFU/mL) on biofilm biomass formation, as assessed by spectrophotometric assay. Values are means ± SDs (n = 6). ***p < 0.001, dynamic vs static, unpaired-*t* test

*S. pseudintermedius* strain DSM 25713 biofilm growth on polystyrene, as assessed by crystal violet assay, under different experimental conditions is summarized in Fig. 1. A similar trend was observed, regardless of the inoculum size considered (10^5^, 10^6^ or 10^7^ CFU/ml). Specifically, although significantly higher (*p* < 0.001) biofilm formation occurred following 24 h-incubation under dynamic conditions compared to static ones, we observed an opposing trend at 48 and 72 h-incubation (*p* < 0.001). Maximum biofilm amount was produced at 10^7^ CFU/ml after 72 h of incubation, while no statistically significant differences were found among the tested inoculum sizes (OD_492_: 2.698, 2.423, and 2.491, at 10^7^, 10^6^, and 10^5^ CFU/ml, respectively; *p* > 0.05). Therefore, an inoculum size of 10^7^ CFU/ml, and static incubation were used for optimal biofilm formation by *S. pseudintermedius* on a polystyrene surface. Since no statistically significant differences in biofilm biomass formation were observed between 48 and 72 h of incubation, we choose to allow the biofilm to grow for 48 h and then expose it to antibiotics for another 24 h in the evaluation of antibiotic activity against preformed biofilm.

### Effects of serum and pH on biofilm formation

**Fig. 2:**
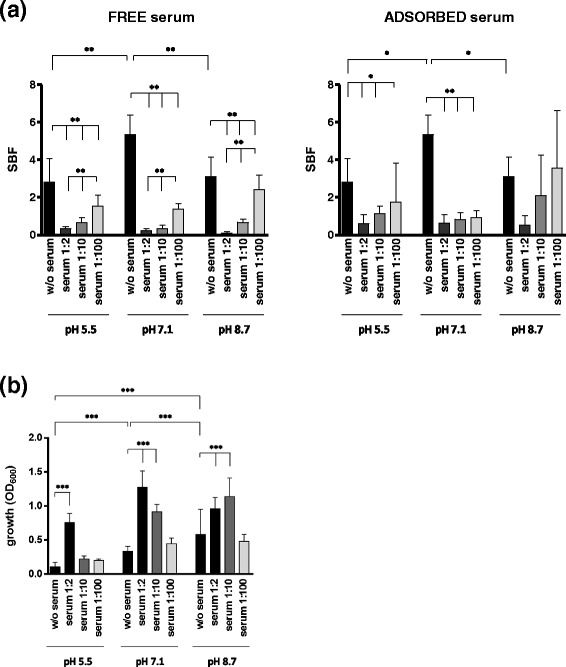
Effect of serum and pH on biofilm formation and growth by *S. pseudintermedius* strain DSM 25713. (**a**) Serum was tested against biofilm formation at various dilutions (1:2, 1:10, and 1:100), as free or adsorbed to polystyrene, under different pH (5.5, 7.1, and 8.7). Control wells contained bacteria but not serum. Biofilm biomass amount was measured by crystal violet assay, then normalized on bacterial growth by calculating the specific biofilm formation (SBF) index (see Materials and Methods). (**b**) The effect of free serum against bacterial growth was assessed by measuring OD_600_ of cell grown in broth following 24 h-incubation. Results are means + SDs (n = 9). *p < 0.05, **p < 0.01, and ***p < 0.001, ANOVA + Newman-Keuls multiple comparison post-test

The combined effects of different serum concentrations and pH values on biofilm formation by *S. pseudintermedius* strain DSM 25713 are summarized in Fig. 2.

In the absence of serum, biofilm formation at pH 7.1 was significantly higher than that obtained at pH 5.5 or 8.7 (SBF: 5.33 ± 1.06 vs 2.82 ± 1.25 and 3.10 ± 1.05, respectively; *p* < 0.05) (Fig. 2a). Furthermore, considering the criteria proposed by Stepanovic *et al*. [20] *S. pseudintermedius* strain DSM 25713 continuously demonstrated a strong capacity to produce biofilm (mean OD_492_ > 0.280), regardless of the pH value considered.

In the presence of serum the ability to form biofilm was significantly affected, regardless of the pH value tested. Specifically, the amount of biofilm was reduced compared to the control, with ranges of 45.3-87.5 %, 74.3-95.5 %, and 21.9-96.5 % at pH values of 5.5, 7.1, and 8.7, respectively (Fig. 2a).

Polystyrene pre-treatment with serum significantly reduced biofilm formation under acidic conditions, but only in the presence of 1:2 and 1:10 serum (reduction vs control: 59.4 and 78.5 %, respectively), and neutral pH (82.7-88.3 %). At pH 8.7, coating with serum did not significantly reduce biofilm formation (Fig. 2a). The anti-biofilm effect was shown to be dependent only upon concentration levels in the case of free serum, regardless of the pH value considered.

As shown by the comparative evaluation of OD_600_ values of supernatant culture that were measured following 24 h-incubation, planktonic growth of *S. pseudintermedius* strain DSM 25713 was significantly enhanced in the presence of serum. This effect was more relevant at pH 7.1 and 8.7, when serum was effective at both 1:2 and 1:10 (Fig. 2b). In the absence of serum, *S. pseudintermedius* strain DSM 25713 growth was pH-dependent, with maximum result at pH 8.7 (OD_600_, mean ± SD: 0.583 ± 0.370 vs 0.337 ± 0.071 and 0.107 ± 0.067 at pH 8.7, 7.1 and 5.5, respectively; *p* < 0.001).

### Susceptibility of planktonic cells to antibiotics

*In vitro* susceptibility of *S. pseudintermedius* strain DSM 25713 planktonic cells to chloramphenicol, gentamicin, cefoxitin, linezolid, rifampicin, tigecycline, tetracycline, and vancomycin is summarized in Table 1.

**Table 1:** *In vitro* antibiotic susceptibility of planktonic and biofilm *S. pseudintermedius* strain DSM 25713 cells. MIC, Minimum Inhibitory Concentration; MBC, Minimum Bactericidal Concentration; MBEC_50_ and MBEC_90_, Minimum Concentrations Eradicating respectively 50 % and 90 % of Biofilm viability, compared to untreated controls (100 % viability). Assays were performed in triplicate and repeated on two different occasions (n = 6). Values are mg/L

Antibiotics	Planktonic cells		Biofilm cells		*C*_max_^a^
	MIC	MBC	MBEC_50_	MBEC_90_	
Chloramphenicol	32	256	128	4,096	16.9 [49]
Gentamicin	0.5	0.5	32	32	5-12 [50]
Cefoxitin	16	32	16	1,024	20 [51]
Linezolid	4	32	4	1,024	21.2 [52]
Rifampicin	0.03	0.5	0.03	0.06	10 [53]
Tigecycline	0.5	8	0.5	64	0.25-2.8 [54]
Tetracycline	0.5	8	0.5	64	2 [55]
Vancomycin	2	2	16	32	25 [56]

^a^Maximum concentration of drug in serum; references are shown in parentheses

MIC values showed that rifampicin is the most active antibiotic among those tested (MIC: 0.03 μg/ml). On the contrary, cefoxitin and chloramphenicol were the least active drugs (MIC: 16 and 32 μg/ml, respectively). The comparative evaluation of MIC and MBC values showed bactericidal activity only for cefoxitin, gentamicin, and vancomycin (MBC/MIC < 4).

### Effects of subinhibitory antibiotic concentrations on biofilm formation

**Fig. 3:**
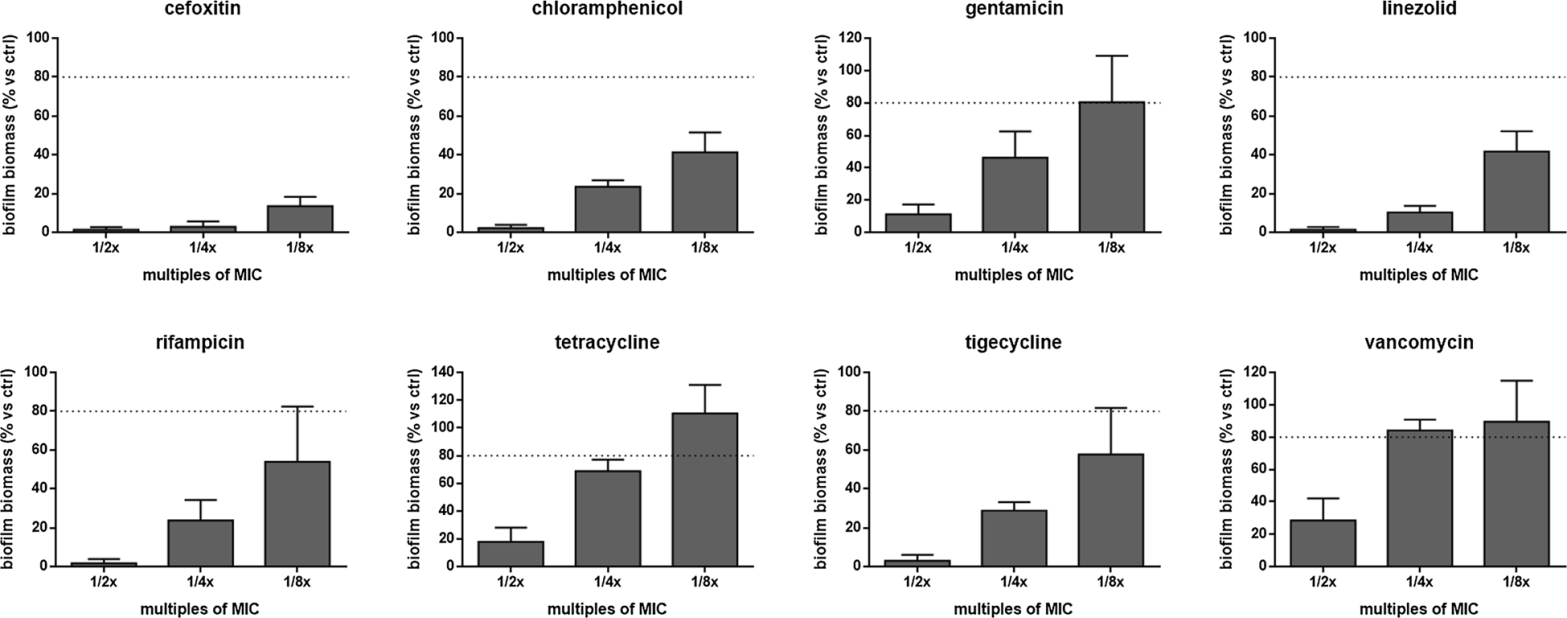
*In vitro* activity of antibiotics at sub-inhibitory concentrations against biofilm formation by *S. pseudintermedius* strain DSM 25713. Biofilm biomass formed during 24 h-incubation was measured, using the crystal violet assay, in the presence of antibiotics at concentrations equal to 1/2x, 1/4x, and 1/8xMIC. Results were plotted as percentage of biofilm biomass formed in the presence of antibiotic, compared to controls (not exposed, 100 % biofilm biomass) (n = 6). The dotted line indicates a reduction in biofilm biomass of at least 20 % vs control (*p* < 0.001, chi-square test)

The effects of antibiotics tested at sub-inhibitory concentrations (1/2x, 1/4x, and 1/8xMIC) against biofilm formation are shown in Fig. 3.

Generally, sub-inhibitory concentrations caused a significant reduction in the formation of biofilm in a dose-dependent manner, although striking differences were observed among the antibiotics tested.

Cefoxitin proved to be the most active antibiotic since at 1/8xMIC it provoked a reduction in biofilm formation of *S. pseudintermedius* strain DSM 25713 that was significantly higher than other antibiotics (% biofilm biomass vs control, 13.5 ± 4.9). Chloramphenicol, gentamicin, linezolid, rifampicin and tigecycline also caused a significant reduction in biomass regardless of the concentrations tested. On the contrary, tetracycline and vancomycin were the least active against antibiotics, showing an inability to affect biofilm formation at concentrations equal to 1/8xMIC or both at 1/4x and 1/8xMIC, respectively.

### Effects of antibiotics on preformed biofilms

**Fig. 4:**
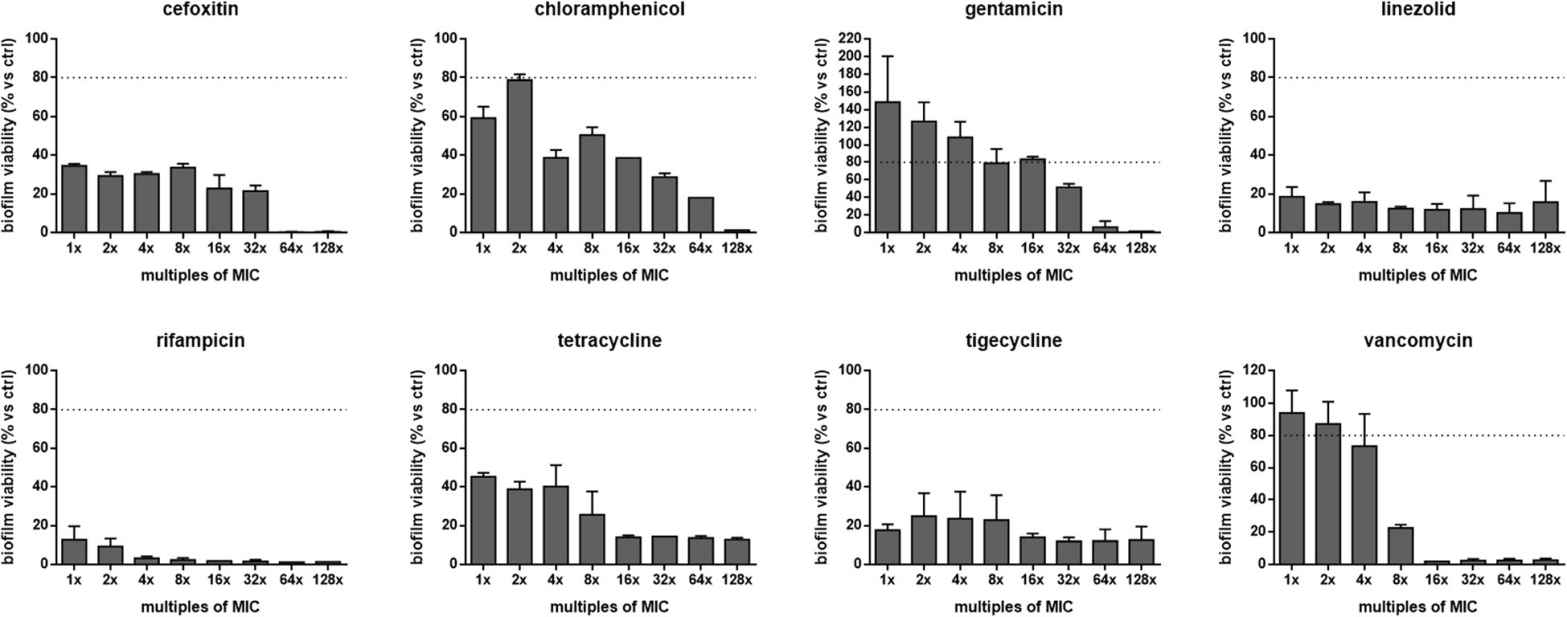
*In vitro* effect of antibiotics against preformed biofilm by *S. pseudintermedius* strain DSM 25713. Biofilms allowed to form following 48 h-incubation were exposed for further 24 h to each antibiotic at concentrations equal or multiple of MIC. Results are expressed as percentage of biofilm’s viability – as assessed by viable colony count - compared to control (unexposed, 100 % viability) (n = 6). The dotted line indicates a reduction in biofilm viability of at least 20 % vs control (*p* < 0.001, chi-square test)

The activity of antibiotics, tested at concentrations equal to or a multiple of MIC, on mature biofilms is summarized in Table 1 and Fig. 4.

The comparative evaluation between MIC and MBEC values indicated that rifampicin is the most active antibiotic against preformed biofilms (MBEC_50_ and MBEC_90_: 1x and 2xMIC, respectively) (Table 1). Other antibiotics showed a reduced activity, although at different extents. In particular, vancomycin showed MBEC_50_ and MBEC_90_ of 8x and 16xMIC, respectively, followed by cefoxitin (MBEC_50_ and MBEC_90_: 1x and 64xMIC, respectively). Chloramphenicol (MBEC_50_ and MBEC_90_: 4x and 128xMIC, respectively), linezolid, tetracycline and tigecycline (MBEC_50_ and MBEC_90_: 1x and >128xMIC, respectively) showed comparable activity. Gentamicin was the least active among the antibiotics tested (MBEC_50_ and MBEC_90_: 64xMIC), even stimulating the production of significantly higher biofilm amounts at 1x and 2xMIC, compared to controls (Fig. 4). All antibiotics exhibited a dose-dependent effect, except for linezolid, rifampicin, and tigecycline. Importantly, none of the antibiotics studied were able to eradicate mature biofilms at the concentrations tested (Fig. 4).

In consideration of the concentrations corresponding to MBEC_90_ values, rifampicin was confirmed to be the most active antibiotic (MBEC_90_: 0.06 μg/ml), followed by vancomycin and gentamicin (MBEC_90_: 32 μg/ml), while chloramphenicol was the least effective (MBEC_90_: 4,096 μg/ml) (Table 1).

### Microscopic analysis of biofilms formed under static and dynamic conditions

**Fig. 5:**
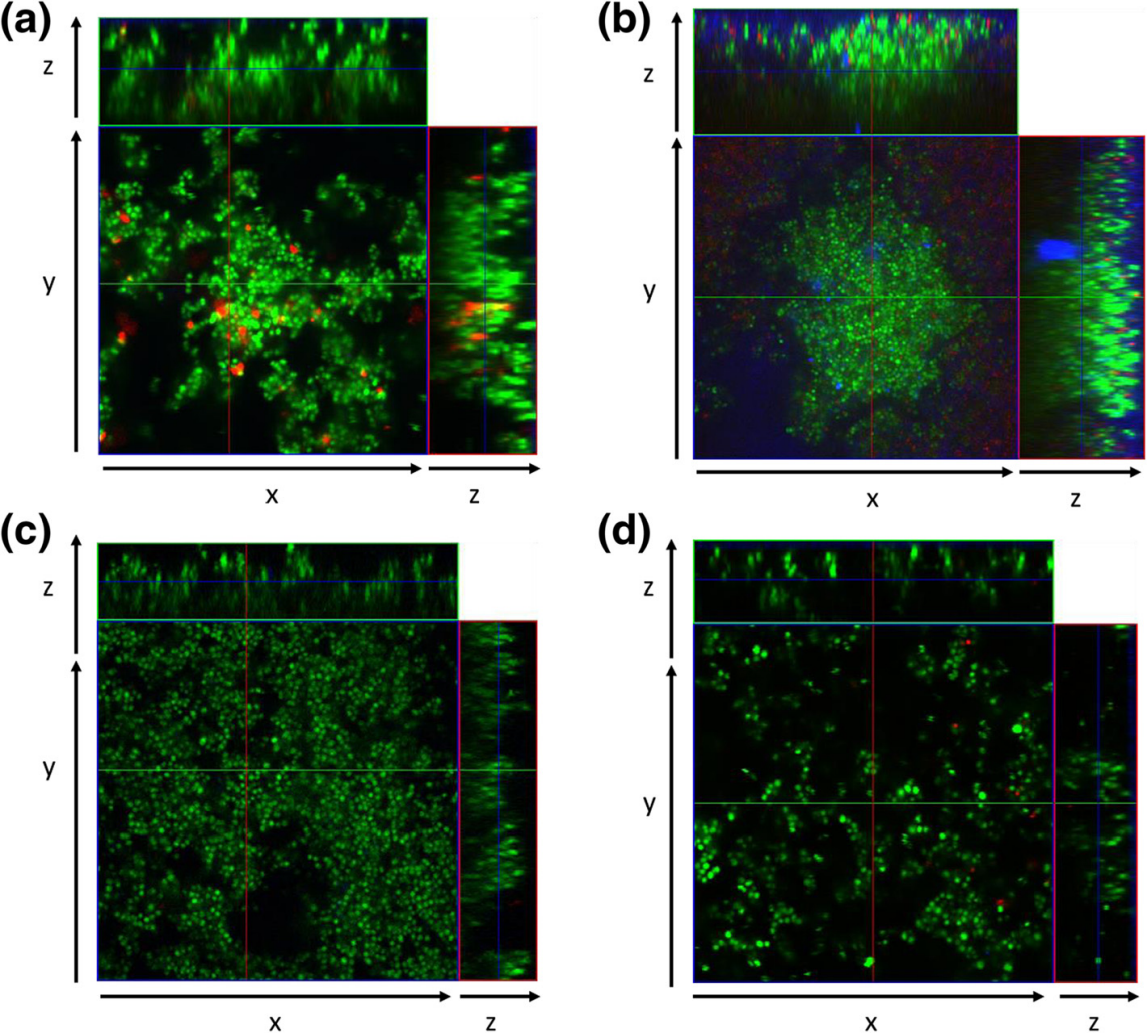
Confocal Laser Scanning Microscopy of biofilm formed by *S. pseudintermedius* strain DSM 25713. Biofilm was allowed to form for 48 h at 37 °C, in absence of serum, under both (**a**) static, and (**b**) dynamic (flow cell chamber) conditions. Static biofilms were further treated for 24 h with increasing gentamicin concentrations (1x-128xMIC). Representative images of biofilm exposed at (**c**) 1x and (**d**) 128xMIC gentamicin are shown. Orthogonal images z are projections of x and y planes, collected within the biofilm as indicated by the green and red lines in the top view. Image capture was set for simultaneous visualization of red (Propidium iodide-stained dead cells), green (Syto-9-stained viable cells), and blue (Concanavalin A-stained EPS) fluorescence. Magnification, x100

Representative CLSM images of biofilm formed by *S. pseudintermedius* strain DSM 25713 are shown in Fig. 5. Under static incubation, *S. pseudintermedius* is able to form a dense biofilm with “mushroom-like” architecture consisting of aggregates and microcolonies that almost completely cover the polystyrene surface. The biofilm formed in flow cell chamber, under dynamic conditions, was shown to be significantly more complex, in terms of thickness and cellularity, compared to the biofilm formed under static incubation (mean thickness, 25.4 vs 14.2 μm, respectively; *p* < 0.05) (Figs. 5a-b).

Corresponding to the results obtained with the viable cell count testing, there were variations in biofilm amount and morphology observed in a dose dependent response to gentamycin. At 1xMIC, biofilm amount was increased, while at 8xMIC and above, there were alterations in the three-dimensional structure of the biofilms seen, and some disruption of established biofilms.

**Fig. 6:**
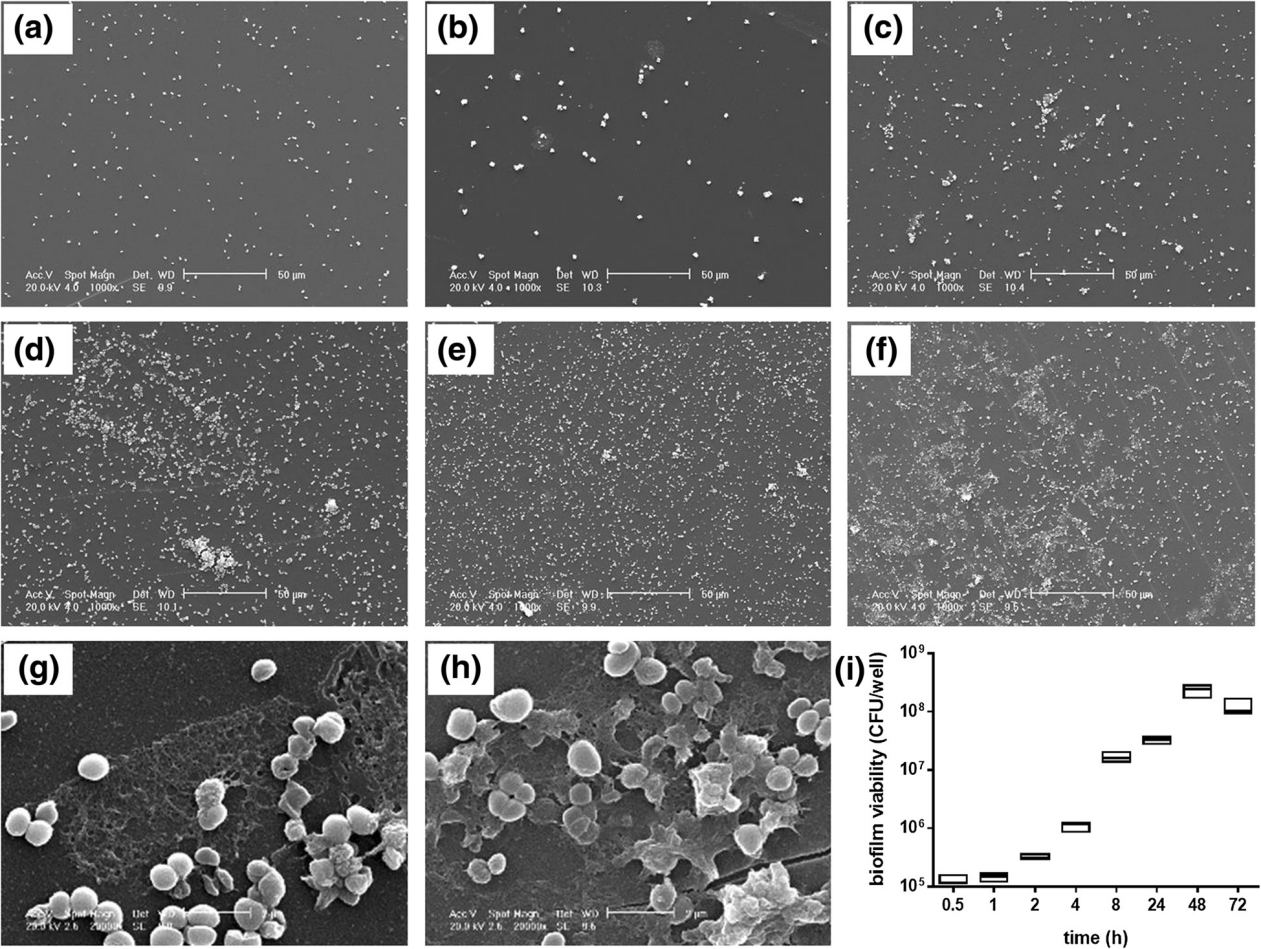
Kinetic of biofilm formation, through 72 h-incubation, by *S. pseudintermedius* strain DSM 25713 onto polystyrene. (**a**-**f**) Representative SEM images of biofilm formation after 1, 4, 8, 24, 48, and 72 h of incubation, respectively. Magnification (x1.000). (**g**, **h**) Magnification (x20.000) of (**e**) and (**f**), respectively. Cocci are surrounded by EPS appearing as an extensive network of filaments stretched among cells and between these and the substratum. (**i**) Kinetic of biofilm formation as assessed by viable count. Maximum, median, and minimum values are shown in each box (n = 6)

SEM analysis was performed to monitor the biofilm formation kinetics throughout 72 h of incubation, and to analyze the morphological characteristics of biofilm (Fig. 6). After only 30 min, single cocci randomly adhered to polystyrene (adhesion phase). After 4 h, early biofilm appeared as small microcolonies, consisting mainly of clustered cells without any evidence of EPS (Fig. 6b). During the maturation phase (8 to 72 h), microcolonies dimensionally increased, covering most of the surface (Figs. 6c-f). In particular, the addition of alcian blue to the fixative solution revealed a significant production of EPS after 48 h, appearing as an extensive network of filaments. EPS covered most of the surface, surrounded biofilm cells and bridged these to the substratum (Figs. 6 g-h).

The kinetics of biofilm formation by *S. pseudintermedius* strain DSM 25713 on the surface of polystyrene wells over 72 h is shown in Fig. 6i. Viable counts confirmed the findings obtained during SEM analysis. In particular, bacteria were shown to attach rapidly, within 1 h of incubation (median: 1.5 x 10^5^ CFU/well), then the biofilm formation increased over time up to 48 h (median: 1.6 x 10^8^ CFU/well).

**Fig. 7:**
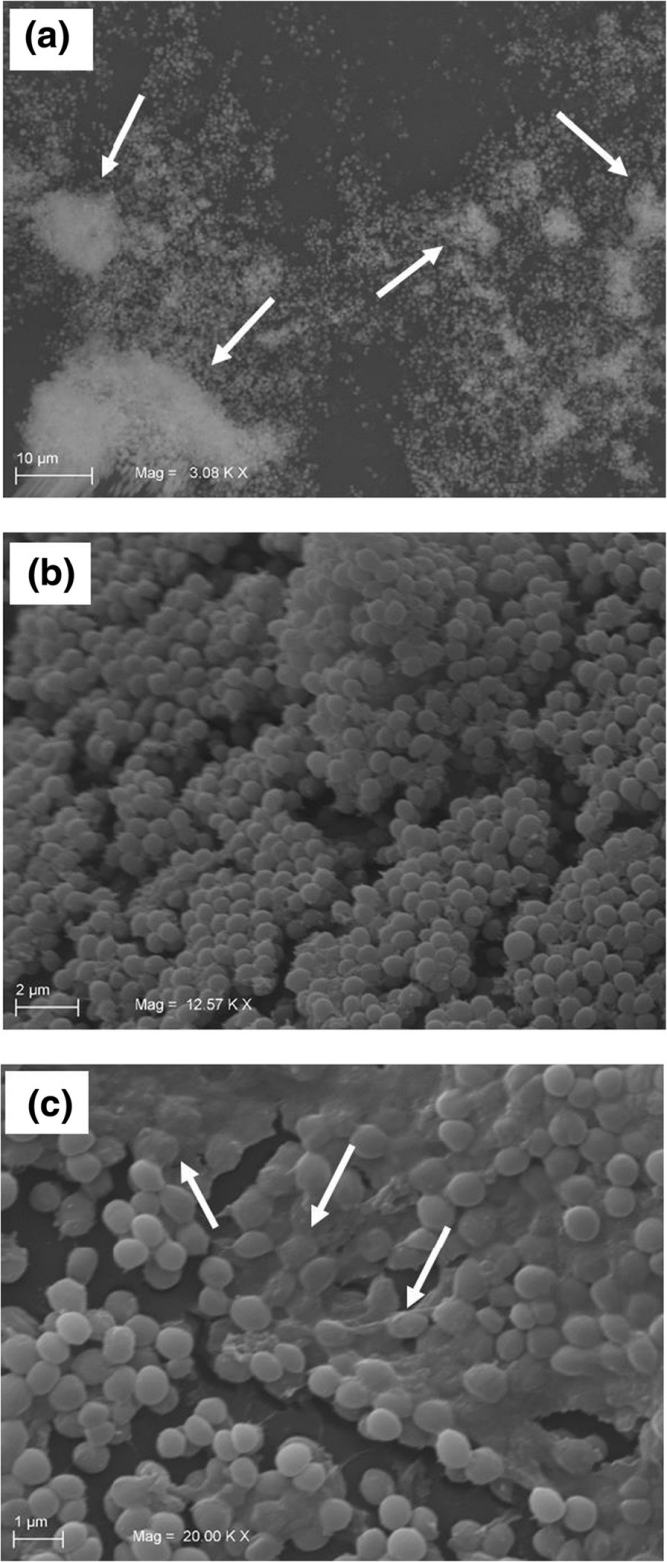
ESEM images of biofilm formed by *S. pseudintermedius* strain DSM 25713 onto polystyrene following 72 h-incubation. (**a**) Biofilm exhibited spatially heterogeneous organization, as suggested by the presence of “mushroom-like” structures (as indicated by arrows). Magnification: x3.000. (**b**, **c**) Multilayered organization with the presence of bacteria under EPS matrix (as indicated by arrows). Magnification: x12.500 and x20.000, respectively

We also performed ESEM to obtain a deeper and more realistic view of the 3D biofilm structure, cell arrangement and matrix shape. ESEM analysis confirmed the heterogeneous architecture of biofilm formed by *S. pseudintermedius* DSM 25713, and disclosed the presence of a highly hydrated extracellular matrix within the surrounding cells (Fig. 7).

## Discussion

Our results demonstrate, for the first time, that human *S. pseudintermedius* has the potential to grow as an antibiotic-resistant biofilm. In fact, confocal and electron microscopy revealed that under static conditions, similarly to wound infections, *S. pseudintermedius* strain DSM 25713 is able to form a well-structured biofilm, consisting of multilayered, mushroom-shaped microcolonies embedded in an abundant amount of EPS matrix, all features highly suggestive of a mature biofilm. SEM observation revealed that EPS, critical for attachment and structural development of mature biofilm [21], forms an extensive network of filaments stretching among cells as well as between cells and the polystyrene surface.

Furthermore, CLSM analysis of *S. pseudintermedius* biofilm formed under dynamic conditions, such as those observed inside a venous or urinary catheter, revealed a more complex ultrastructure compared with that observed under static conditions, with significant increases in both cellularity and thickness. These findings show the potential for *S. pseudintermedius* to cause an implant-associated infection, which is consistent with previous reports of *S. pseudintermedius* causing infections associated with intravascular devices (cardiac devices, catheters) [3–5].

The ability of bacteria to form biofilms has recently been demonstrated as a cause for the chronicization of wound infections [22–25]. Various factors may modulate *in vivo* biofilm development at a wound infection site [26–29]. For example, serum proteins (i.e. fibrinogen and albumin) deposited onto host tissues provide receptor binding sites for bacterial adhesion and biofilm formation [26–28]. In addiction, wound exudate pH, modulated during infection and healing processes, affects bacterial growth and density in biofilm populations [29]. Therefore, the present study examined the ability of the *S. pseudintermedius* strain DSM 25713 to form biofilms in the presence of serum and at different pH values, conditions relevant to the wound environment.

Our results show that the presence of human serum negatively affects *S. pseudintermedius* biofilm formation, although it is worthy to note that the ability to form biofilm was retained even in the presence of serum concentrations well above those observed at the site of infection. Our findings are in agreement with those found for *S. aureus* and *P. aeruginosa* [30, 31], but discordant with studies focused on other Gram-positive bacteria - including *Streptococcus mitis*, *S. aureus*, and coagulase-negative staphylococci – whose adherence was not significantly inhibited by serum [32–34].

The anti-biofilm effect of serum is not due to antibacterial effect since, according to previous findings obtained for *P. aeruginosa* [30], serum significantly promoted the growth of *S. pseudintermedius*, regardless of the pH levels tested.

Precoating polystyrene resulted in a significant decrease in biofilm formation, when compared to the uncoated controls, even in the presence of 1 % serum, thus suggesting that serum components, such as albumin, adsorbed on the substratum surface prevent bacterial attachment by acting as a physical barrier between bacteria and the substratum, or by making the surface less hydrophobic. In particular, the anti-adhesive effect of human serum albumin seems related to the competitive binding of this protein to an accretion surface or bacterial cells [35]. Further studies are warranted in this regard.

The reduction of *S. pseudintermedius* attachment and biofilm formation that we observed in the presence of free serum also suggests that serum might inhibit biofilm formation by additional mechanisms other than coating the surface. As previously observed for *P. aeruginosa* [30], particular components of serum could directly interact with *S. pseudintermedius* thus inhibiting biofilm formation.

Knowledge of the anti-biofilm activity of antibiotics is critical for the management of biofilm-related infections, such as chronic wounds. In the present work, we selected gentamicin, cefoxitin, linezolid, rifampicin, tigecycline and vancomycin because of their relevance in the prevention and treatment of staphylococcal infections, particularly those caused by methicillin-resistant *S. aureus* (MRSA). The older drugs chloramphenicol and tetracycline were also considered, since older antibiotics are frequently being re-evaluated for treatment of multi-drug resistant and biofilm-based infections, due to the decrease in development of novel antimicrobials [36].

Since the bacterial adhesion to a surface is a critical prerequisite for biofilm formation, we first investigated the prophylactic potential of the selected antibiotics by assessing the effect of sub-inhibitory concentrations against both adhesion and biofilm biomass formation. All antibiotics showed both anti-adhesive and anti-biofilm effect, although to different extents. Particularly, cefoxitin exhibited the strongest activity, demonstrating the ability to reduce biofilm biomass formation more than 90 % regardless of the tested concentration. On the contrary, the smallest effect was observed for vancomycin, since it was ineffective against biofilm formation even at 1/8x and 1/4xMIC. Antibiotic concentrations effective against biofilm formation were not active against planktonic growth, and no correlation was found between the ability to kill planktonic cells and the activity on biofilm formation. Together, these findings suggest that the tested antibiotics interfere with *S. pseudintermedius* biofilm formation by mechanisms other than direct antimicrobial activity.

Chronic infections, including wounds and implant-associated infections, often persist despite antibiotic therapy and the innate and adaptive immune and inflammatory responses of the host because due to the presence of biofilm-growing bacteria [37]. Clinically used antibiotics and their dose regimens were in fact classically developed to treat infections due to the presence of planktonic bacteria, therefore they are ineffective in the eradication of biofilm-based infections at the same doses. To evaluate the antibiotic activity against preformed biofilm by *S. pseudintermedius* strain DSM 25713, MBEC of each antibiotic was measured following 24 h-exposure of 48 h-old biofilms to bactericidal concentrations. Overall, MBEC values were greater than CLSI-suggested planktonic MIC breakpoint for resistance. Comparative evaluation of MBEC_90_/MIC ratio - an important parameter for choosing the antibiotic in the treatment of biofilm-associated infections - indicated rifampicin as the most active antibiotic (MBEC_90_/MIC: 2), in agreement with previous *in vitro* and *in vivo* studies focused on *S. aureus* biofilm [38, 39]. Other antibiotics tested showed a significantly reduced activity against preformed *S. pseudintermedius* biofilm as suggested by MBEC_90_ values ranging from 16xMIC (vancomycin) to at least 128xMIC (linezolid, tigecycline, chloramphenicol, and tetracycline). In agreement with our findings, Leite *et al*. [40] found that rifampicin was more active than linezolid against *S. epidermidis* biofilms, while Parra-Ruiz *et al*. [41] observed that linezolid was not bactericidal against mature biofilms formed by *S. aureus*. Our results with *S. pseudintermedius* biofilms are consistent with the findings of vancomycin antibiotic-lock resistant catheter-associated *S. pseudintermedius* bacteremia described by Chuang *et al*. [4], being due to the presence of resistant biofilms.

The clinical relevance of our results is even more evident if peak serum antibiotic concentrations are considered. With the exception of rifampicin, none of the tested antibiotics would have been able to eradicate biofilm even when used at multiples of achievable serum levels from currently recommended dosages. In particular, chloramphenicol, tigecycline, cefoxitin, and linezolid required from 23 to 256 times the maximum attainable concentration in serum to achieve 90 %-inhibition of biofilm viability. The relative lack of efficacy of linezolid and tigecycline in eradicating *S. pseudintermedius* cells embedded in biofilm is consistent with prior *in vitro* studies concerning staphylococcal biofilms [42, 43], raising a special clinical concern since these antibiotics are used as the "treatment of last resort" against potentially life-threatening MRSA infections that are sometimes not treatable with any other antibiotics.

The mechanisms of biofilm resistance are likely multifactorial and vary according to the considered specie, and remain still unclear [44]. Although the present work was not focused on mechanisms underlying the inherent antibiotic-resistance of *S. pseudintermedius* biofilm, the complex biofilm structure, as revealed by microscopic analysis, might play a role in this regard by physically/chemically sequestering the antibiotic, thus delaying its penetration through the biofilm.

Gentamycin, whose MBEC_90_ is 2.6 to 6.4 times higher than peak serum, may be an example of this, where interactions between the positively charged antibiotic and the negatively charged components of EPS likely are responsible for preventing ready diffusion of the antimicrobials through the biofilm matrix to the bacteria [44]. Despite the potential inherent resistance of biofilms to aminoglycosides, the increased concentrations afforded by topical therapy [7, 45], or treatment of wounds where aminoglycosides are relatively concentrated [46], may still allow for the successful eradication of biofilm infections.

## Conclusions

Some clinical implications can be drawn on the basis of our results. First, while *S. pseudintermedius* is a commensal in dogs, our results show significant concern for the organism as a pathogen in people, especially when associated with temporary or permanent implants [3–5]. Second, serum reduces, but does not prevent, *S. pseudintermedius* biofilm formation at concentrations measured in infected wound exudate. Third, *S. pseudintermedius* is able to form mature biofilm inherently resistant to antibiotics at concentrations well above those observed in serum, including linezolid, tigecycline, and vancomycin which are commonly considered as “last resort antibiotics” against methicillin-resistant staphylococci. This is particularly relevant in chronic GvHD patients where skin wound infections account for the majority of deaths [47]. Fourth, *in vitro* models relevant to the *in vivo* situation are needed for adequately assessing antibiotic activity in the case of biofilm-related infections, such as for wounds. Using our model, rifampicin was measured to be the most effective antibiotic against *S. pseudintermedius* strain DSM 25713 biofilms, however clinical use of rifampicin as a sole agent should be appreciated cautiously due to the rapid selection of rifampicin-resistant mutants [48].

The data presented here are *in vitro* results and, therefore, cannot completely explain or represent *S. pseudintermedius* biofilm-related infections. Both *in vitro* and *in vivo* models representative of the wound environment are required to gain new insights into the mechanisms underlying bacterial adhesion and biofilm formation aimed at designing new therapeutic strategies.

## Abbreviations

ICD: Implantation of a cardioverter-defribrillator device
GvHD: Graft-versus-Host Disease
DSM: Deutsche Sammlung von Mikroorganismen und Zellkulturen, GmbH)
EPS: Extracellular polymer substance
TSB: Trypticase soy broth
MHA: Mueller-Hinton Agar
OD: Optical density
MBEC: Minimum biofilm eradication concentration
CAMHB: Cation-Adjusted Mueller-Hinton Broth
SEM: Scanning electron microscopy
ESEM: Environmental-SEM
CLSM: Confocal laser scanning microscopy
SBF: Specific biofilm formation
MRSA: Methicillin-resistant *Staphylococcus aureus*
